# ‘Raising the Pulse’: The environmental, nutritional and health benefits of pulse‐enhanced foods

**DOI:** 10.1111/nbu.12601

**Published:** 2023-01-17

**Authors:** Julie A. Lovegrove, Donal M. O'Sullivan, Paola Tosi, Elena Millan, Lindsay C. Todman, Jacob Bishop, Afroditi Chatzifragkou, Miriam E Clegg, John Hammond, Kim G. Jackson, Philip J. Jones, Stella Lignou, Anna L. Macready, Yvonne McMeel, Jane Parker, Julia Rodriguez‐Garcia, Paul Sharp, Liz J. Shaw, Laurence G. Smith, Matt Tebbit

**Affiliations:** ^1^ Hugh Sinclair Unit of Human Nutrition, Department of Food and Nutritional Sciences University of Reading Reading UK; ^2^ Institute of Cardiovascular and Metabolic Research University of Reading Reading UK; ^3^ Institute of Food, Nutrition and Health University of Reading Reading UK; ^4^ School of Agriculture, Policy and Development University of Reading Reading UK; ^5^ Department of Agri‐Food Economics and Marketing University of Reading Reading UK; ^6^ Department of Food and Nutritional Sciences University of Reading Reading UK; ^7^ Department of Nutritional Sciences King College London London UK; ^8^ Department of Geography and Environmental Science University of Reading Reading UK; ^9^ Catering University of Reading Reading UK

**Keywords:** consumer attitudes and behaviour, environmental impact, faba beans, flour, iron status, pulses, sustainable food systems, white bread

## Abstract

Diet is a key modulator of non‐communicable diseases, and food production represents a major cause of environmental degradation and greenhouse gas emissions. Yet, ‘nudging’ people to make better food choices is challenging, as factors including affordability, convenience and taste often take priority over the achievement of health and environmental benefits. The overall ‘Raising the Pulse’ project aim is to bring about a step change in the nutritional value of the UK consumers' diet, and to do so in a way that leads to improved health and greater sustainability within the UK food system. To achieve our objectives, UK‐specific faba bean production systems that optimise both end users' diets and environmental and economic sustainability of production will be implemented in collaboration with key stakeholders (including industry, the retail sector and government). Palatable faba bean flours will be produced and used to develop ‘Raising the Pulse’ food products with improved nutritional profile and environmental value. Consumer focus groups and workshops will establish attitudes, preferences, drivers of and barriers to increased consumption of such products. They will inform the co‐creation of sensory testing and University‐wide intervention studies to evaluate the effects of pulses and ‘Raising the Pulse’ foods on diet quality, self‐reported satiety, nutritional knowledge, consumer acceptance and market potential. Nutrient bioavailability and satiety will be evaluated in a randomised‐controlled postprandial human study. Finally, a system model will be developed that predicts changes to land use, environment, business viability, nutrition and human health after substitution of existing less nutritionally beneficial and environmentally sustainable ingredients with pulses. Government health and sustainability priorities will be addressed, helping to define policy‐relevant solutions with significant beneficial supply chain economic impacts and transformed sustainable food systems to improve consumer diet quality, health and the environment.

## INTRODUCTION

**FIGURE 1:**
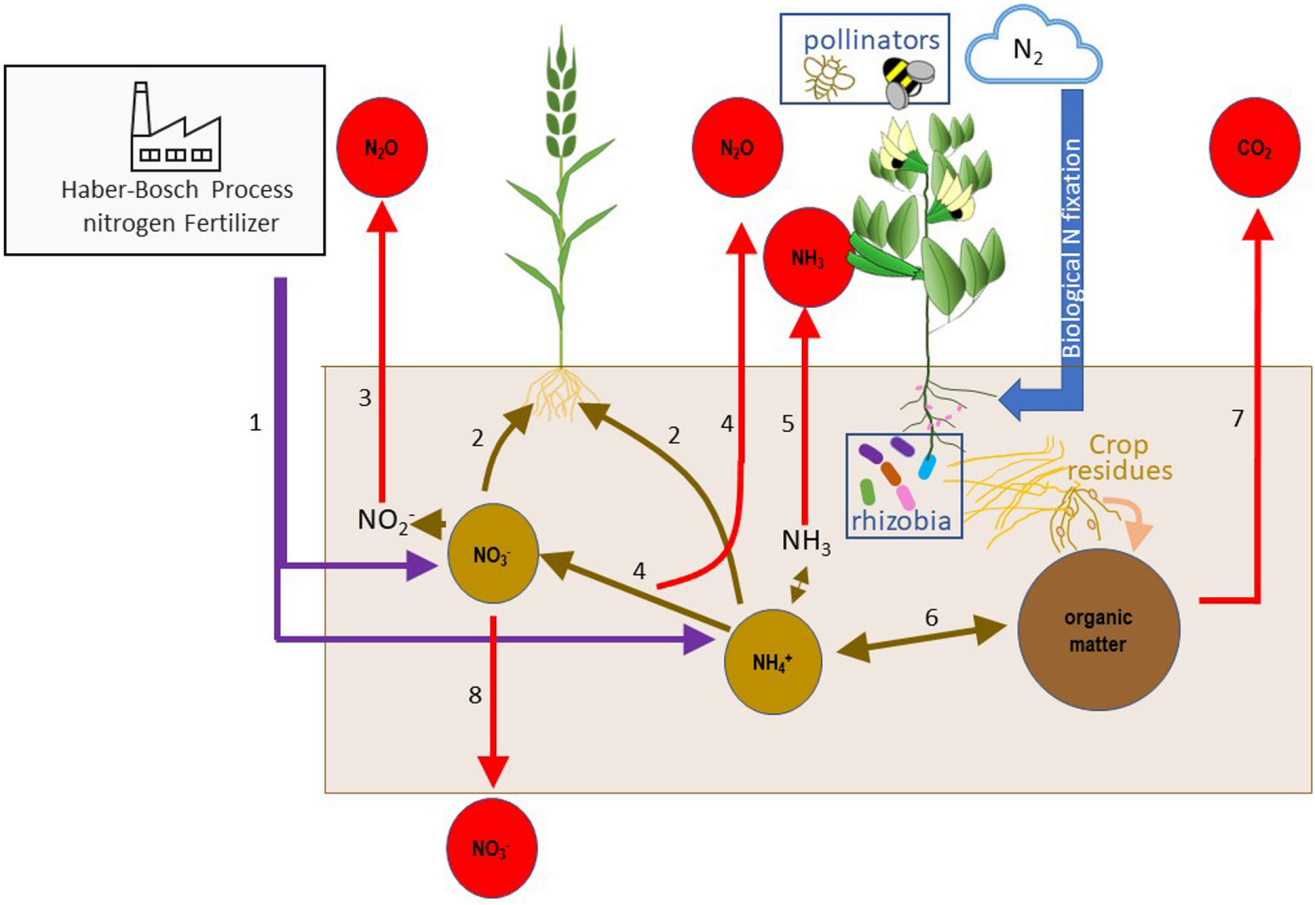
Nitrogen and soil organic matter dynamics in faba bean (in rotation with wheat) showing the properties to be determined in the ‘Raising the Pulse’ field trials: Nitrogen input through biological nitrogen fixation in faba bean (blue arrow); soil available nitrogen and organic matter pools (brown circles); gaseous (CO_2_, N_2_O, NH_3_) and leaching (NO_3_^−^) losses (red circles); Rhizobial and pollinator abundance and diversity (boxes). Contributing industrial and biogeochemical processes are indicated by numbered arrows: 1. Fertiliser N input (in wheat) feeding soil nitrate and ammonium pools; 2. Root uptake; 3. Denitrification and 4. Nitrification resulting in the production of N_2_O; 5. Ammonia volatilization; 6. N mineralization/immobilisation supplying plant‐ available N pools; 7. Soil (microbial) respiration; 8. Nitrate leaching

Globally, non‐communicable diseases cause the greatest mortality, although this can be largely prevented by dietary change. Legumes have health benefits and their underconsumption has been associated with loss of almost a million Disability Adjusted Life Years (DALYs) worldwide (Fulgoni et al., 2018). Cardiovascular diseases are an important contributor to lost DALYs, with diets higher in pulses shown to lower circulating risk markers, including blood lipids (Ferreira et al., 2021). Pulses are a subset of the nitrogen‐fixing Leguminosae family (legumes) and produce edible protein‐rich seeds that are readily harvested, stored and traded as dry seed. They are a vital component of a balanced diet, particularly plant‐based diets, due to their high‐protein content and general richness in soluble fibre, folate, iron and zinc as compared with the staple cereals (Hall et al., 2016; Rebello et al., 2014). Furthermore, when compared with other nutrient‐dense foods like quinoa, pulse production has greater inherent sustainability due to the ability of pulses, in cooperation with symbiotic rhizobia bacteria, to biologically fix nitrogen from the atmosphere (rather than relying on fertilisation with artificial nitrogen fertilisers synthesised with high‐carbon footprint) to form edible protein. In addition, because legume‐fixed nitrogen is less likely to be lost from soil through gaseous emissions or leaching than fertiliser nitrogen (Stagnari et al., 2017), pulse crops also generally have lower greenhouse gas (i.e., nitrous oxide) emission and lower environmental impacts than non‐legumes (Figure 1). Biologically, fixed nitrogen may also transfer to subsequent non‐legume crops in rotation, offsetting the need for synthetic nitrogen fertiliser use (Preissel et al., 2015).

There is considerable diversity in pulses, through variation in size, texture, taste, cooking time, exact nutritional specification and adaptation to specific growing environments. In the UK context, one pulse, the faba bean, stands out above all others due to its potential for transformational dietary change and environmental sustainability. Amongst grain legumes, faba bean has the highest yield potential (Cernay et al., 2016) and nitrogen‐fixation rates (Baddeley et al., 2013) in the United Kingdom and globally, while providing valuable floral resources for a diversity of wild pollinating insects (Bailes et al., 2018). As it can be sown as a winter or spring crop and can be harvested for a long period following maturation, it is also the most flexible pulse for a diversity of farming systems and end uses. These innate characteristics mean that faba bean is regarded as a highly suitable restorative ‘break’ crop in many arable rotations. In terms of health benefits, faba bean has a high‐protein content (28%–29%), is micronutrient‐dense and higher intakes have been reported to lower blood cholesterol. When consumed in the form of faba bean‐enhanced flour in pasta, it was reported to improve post meal satiety (Greffeuille et al., 2015), glycaemia and blood pressure (Chan et al., 2019). Faba beans have promisingly high levels of iron (up to 21.3 mg/100 mg dried weight; Labba et al., 2021) and may also help to reduce the risk of iron deficiency anaemia, the most common diet‐related deficiency in the United Kingdom (PHE & FSA, 2020). It has long been known that faba bean seeds may contain significant levels of the anti‐nutritional compounds vicine and convicine, ingestion of which can trigger favism, a form of haemolytic anaemia, in genetically pre‐disposed individuals deficient in glucose‐6‐phosphate dehydrogenase (approx. 4% of the human population; Luzzatto & Arese, 2018). However, low‐vicine varieties that pose no consumer risk have already been released in the United Kingdom (PGRO Pulse Descriptive List, 2021). The recent discovery of the vicine biosynthetic pathway (Björnsdotter et al., 2021) paves the way for vicine‐free cultivars and the vicine content of any pulse‐enhanced foods or food products developed in the project will be carefully monitored. In consideration of all the above features, we consider that the faba bean represents a safe and sustainable UK pulse with great potential to improve overall diet quality and consumer health.

In line with the Eatwell Guide guidance (Scheelbeek et al., 2020) and the health and environment benefits of pulses, the overarching aim of this project is to ‘raise the pulse’ content of UK diets. Dietary and consumer aspects of the ‘Raising the Pulse’ project will explore attitudes to, preferences for, and role of pulses in people's diets, with a specific focus on faba beans. Making transformative change with sufficient urgency, on a scale with global environmental impact and potential to benefit human health, will require the engagement of all population groups, including disadvantaged groups, who often lack the financial resources and/or knowledge to make major dietary changes (White, 2017). The premise of this project is to adapt the food system to permit food substitutions that benefit both human and environmental health without requiring major adjustments in terms of taste or diet and with minimal extra cost. Ultimately, the ‘Raising the Pulse’ project aims to make it easy for people to consume more UK‐grown pulses, rather than nutritionally inferior and environmentally more costly components of the diet, such as refined breads and processed meats. Over 96% of the UK population consume bread, 90% of which is white, with a higher proportional spend on bread by lower socio‐economic groups (Kantar, 2020; Lockyer & Spiro, 2020). Inclusion of sustainable UK‐grown faba bean in mass‐market white bread as the exemplar product would lead to a step change in pulse consumption and nutrient intake in both receptive and recalcitrant population groups. To achieve this aim, our production, processing, nutritional and consumer science work will focus on routes to creating bread with enhanced nutritional and environmental benefits and high‐consumer acceptability. A system‐level understanding of this exemplar food substitution would make a measurable difference to nutrient intake, health and environment through the enhancement of a mass‐market food product and offers a strategy also applicable to other elements of the UK household food basket.

Our hypothesis is that the inclusion of sustainably produced faba beans in the UK diet, by successful part‐substitution of cereals and soy in mass market staple foods such as bread, will lead to substantial simultaneous gains in dietary quality and public health, thus reducing micronutrient deficiencies (e.g., iron deficiency anaemia) and chronic disease risk, and also bringing positive land use change, and environmental impact mitigation.

**FIGURE 2:**
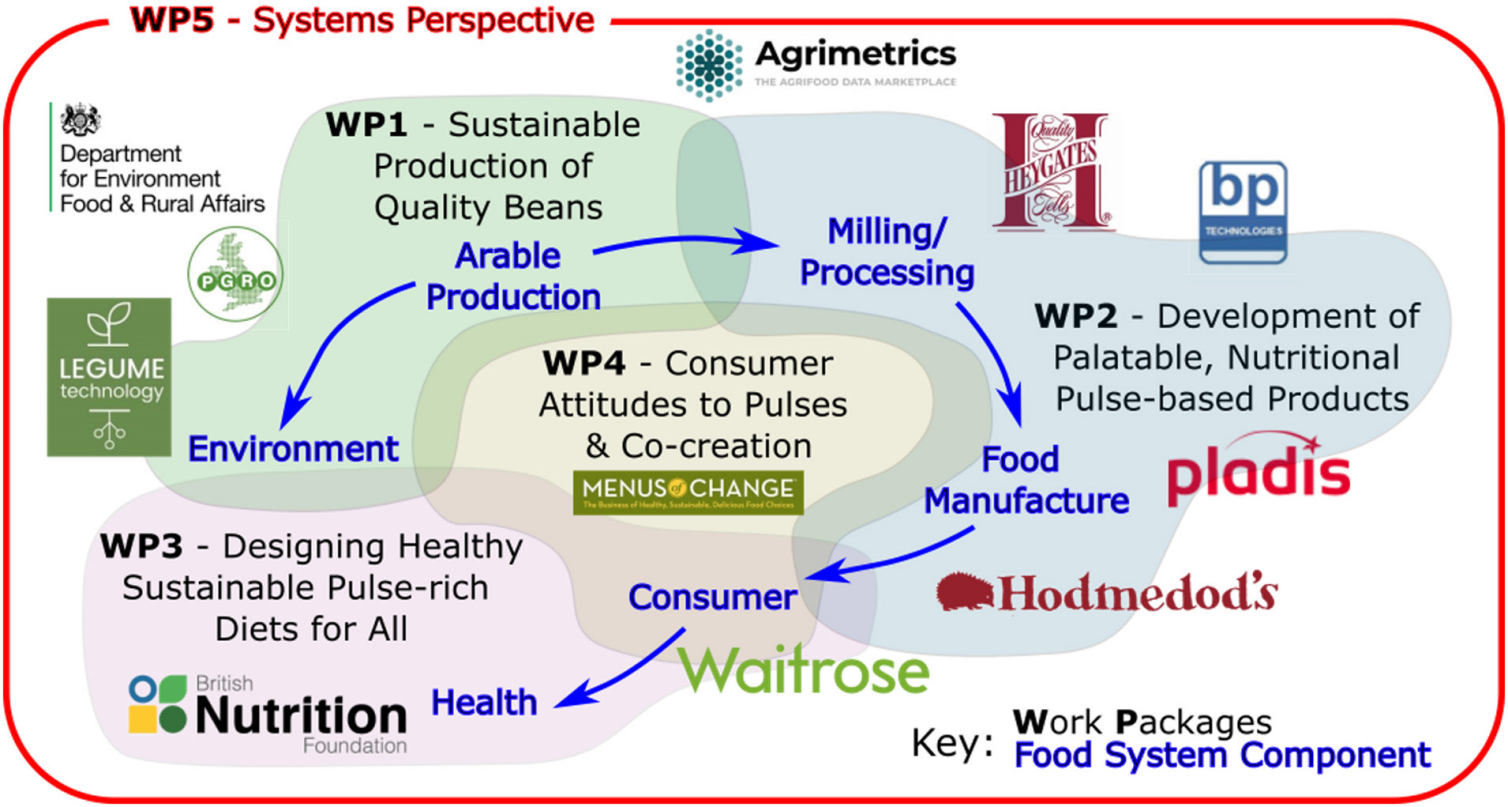
‘Raising the Pulse’ schematic showing highly interactive, multi‐disciplinary work packages (WPs) organised in a consumer‐centred strategy, with involvement of stakeholders in all parts of the project

The ‘Raising the Pulse’ project offers a solution to these challenges and will tackle a series of related obstacles to widespread use of UK‐grown pulses in mainstream foods in a coordinated, holistic way with a multi‐disciplinary team able to introduce cutting edge innovation in each component of the food system. As shown in Figure 2, working and co‐creating with consumers, gathering insight into their attitudes and behaviour towards pulses, particularly faba beans, as foods and food ingredients, as well as with environmental and health food choice influencers, will be at the heart of the project, interacting with and informing all other project aspects. We will build on our prior work on faba bean quality and sustainability to co‐optimise environmental footprints of production and raw material quantity and quality. We will optimise the pre‐processing, milling and baking steps needed to create faba bean flours with improved nutritional profile and technological properties, and prototype pulse‐enhanced ‘Raising the Pulse’ breads and other food products with minimal flavour change. The nutritional benefits, bioavailability of key nutrients (including iron, satiety and impact on metabolic and CVD risk biomarkers of ‘Raising the Pulse’ breads) will be determined alongside the generation of consumer knowledge throughout the different phases of the project work. Finally, a unique and novel system model will be developed capturing main dynamics and interactions that drive nutritional, economic and environmental performance of the system as a whole.

**FIGURE 3:**
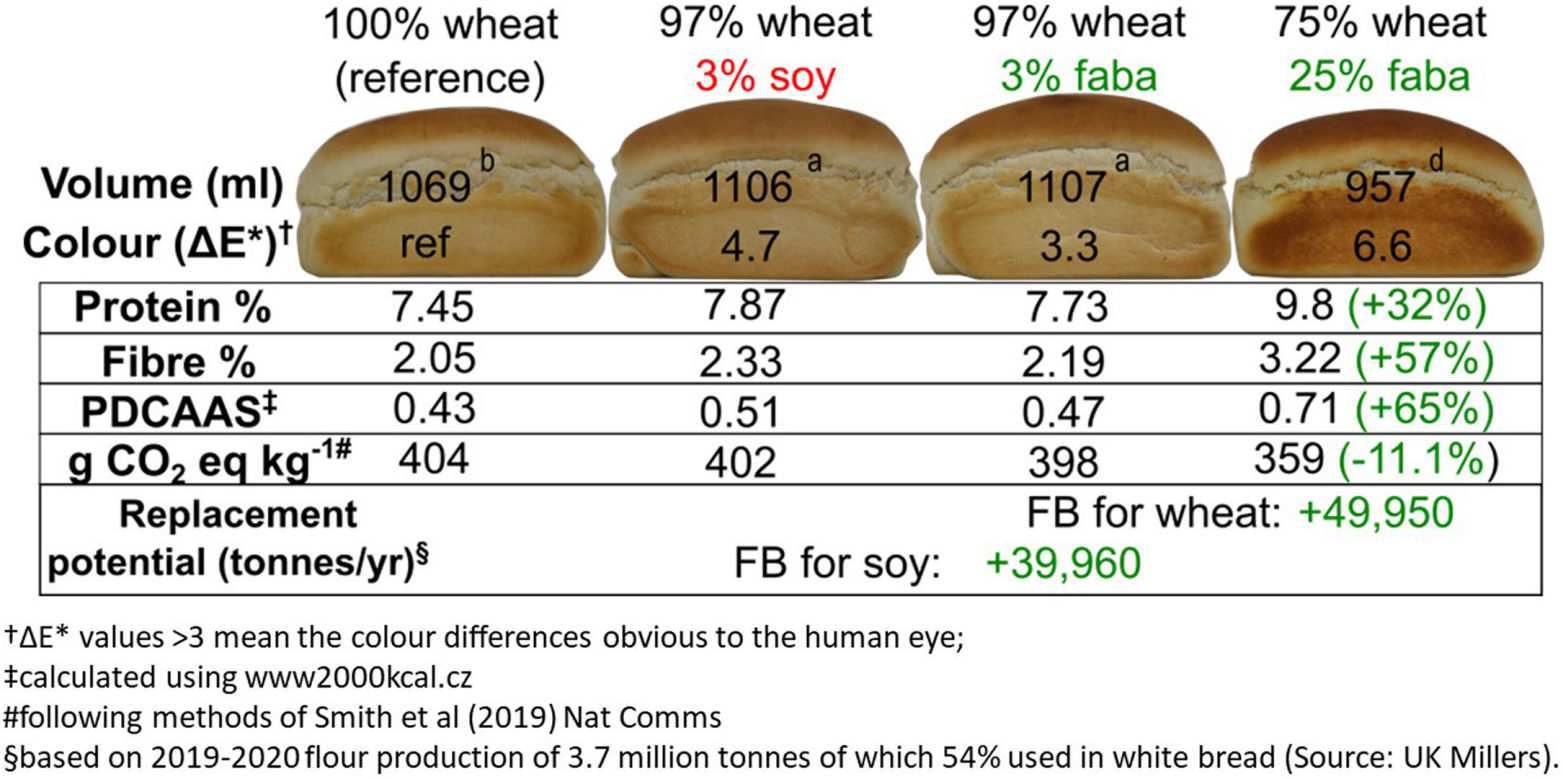
Preliminary findings on the impact potential of composite bread

To demonstrate feasibility and establish a baseline for this project, the ‘Raising the Pulse’ team carried out laboratory pilot baking of composite faba‐wheat bread loaves and conducted preliminary composition, sensory and environmental impact assessment. The first aim of the pilot study was to test whether faba bean flour could replace the soy flour typically used as an improving agent in the production of a typical white bread loaf. The nutritional composition of wheat, soy and faba flours is shown in Table 1. Full‐fat, enzyme‐active soya flour is commonly used at about a 3% inclusion rate as its lipoxygenase activity both bleaches the flour and helps gluten development and hence loaf volume by oxidising the dough (Cauvain, 2020). In fact, where 3% soy flour was replaced with an equal weight of faba bean flour, an equal rise in loaf volume and similar colour was obtained suggesting this minor ingredient substitution to be highly feasible (Figure 3). Complete adoption of faba bean flour as the sole improver replacing imported soy in UK industrial bread would likely generate demand for 40 kt of faba beans per annum over and above current production. However, substitution of one minor pulse ingredient (soy) with another (faba) had a minimal effect on overall nutritional composition, so the second aim of the pilot study was to assess the consequences of more substantial ingredient changes, where not only the soy improver, but a good part of the wheat flour is substituted. As illustrated in Figure 3, faba bean flour substitution rates of 25%, produced loaves with a noticeably darker colour and a reduced loaf volume and springiness, trade‐offs which will be addressed in the course of the project. However, no modifications were required to the baking process and minimal sensory property changes were reported. Total fibre and protein content increased by 57% and 32%, respectively, relative to the reference 100% wheat loaf, and, crucially, the protein digestibility score (PDCAAS) shifted from 0.43 to a more favourable 0.71 (perfectly digestible animal protein having a PDCAAS of 1). In terms of environmental impact, if 75 wheat: 25 faba bean composite bread reached even a 10% market share in the United Kingdom, this would generate demand for a further 50 kt of faba bean per annum, driving widespread, favourable land use change and a concomitant 11% drop in CO_2_ (equivalent) emissions per loaf compared to production of the reference (wheat‐only) loaf. Once a quality‐oriented supply chain able to deliver products of specified quality and traceable environmental footprint has been established for faba bean‐enhanced bread, further transformative change involving pulse‐enhancement of a range of staple and convenience foods, including snacks, meat replacements, and drinks will become possible.

**TABLE 1:** Composition of selected nutrients for white wheat, soya and faba bean flour

Per 100 g	Wheat flour^a^	Soy flour^a^	Faba bean flour^b^
Energy (kJ/kcal)	1504 (353)	1711 (409)	1407 (331)
Protein (g)	11.3	34.3	28.0
Total carbohydrates (g)	79.2	18.7	54.7
Total fats (g)	1.2	22.4	1.6
Dietary fibre (AOAC) (g)	3.3	18.0	13.8^c^
Iron (mg)	1.9	8.4	5.5

Abbreviations: AOAC, Association of Analytical Chemists; NSP, non starch polysaccharides.

^a^ Nutrient composition taken from Finglas et al. (2015).

^b^ Nutrient composition taken from Miller et al. (2019).

^c^ Total dietary fibre.

## WORK PACKAGE 1 – SUSTAINABLE PRODUCTION OF QUALITY BEANS

The objective of this work package is to quantify variation in the productivity, quality and environmental benefits amongst top‐yielding faba bean varieties (genotypes) and those low in vicine (Björnsdotter et al., 2021), which is the main trigger of favism, or phytate. Trials will be conducted across the University of Reading and partner farms (× Environment) with on‐farm trials expanding the testing of promising genotype × management combinations to a range of farming systems and environments.

1. *Optimising Faba Bean Footprints*. There are major gaps in UK faba bean agronomy that we will address through a uniquely comprehensive field evaluation of Genotype × Management × Environment (G × M × E) interactions in the efficient production of diverse bean varieties considered most suitable for use as raw materials for milling. Management trials will follow conventional practices (appropriate fertiliser and mineral treatments will be applied to meet recommended levels following soil analysis) and test the effect of a rhizobial stimulant at plot level over 3 years at University of Reading Sonning Farm. The trials will also assess the effects of genotype and management in an agricultural rotation by measuring nutrient transfer and performance of a first wheat crop following the bean crop.
2. *Comprehensive Sustainability Metrics* will be evaluated from the field trials described above. Sampling will focus on measuring the prominent ecosystem services provided by legumes: biological nitrogen‐fixation and wider regulation of nitrogen and soil organic matter cycling (influencing soil carbon and nitrogen storage, gaseous emissions and other loss processes); regulation of rhizobial and insect pollinator biodiversity. We will also measure how biodiversity supports crop yield production. Diversity and traits of faba bean symbiotic rhizobia in UK soils will provide valuable information on the need for, and potential optimisation of rhizobial inoculation. The role of genotype and management in the provision of resources for pollinating insects will be assessed by floral nectar measurements to estimate the mass of sugar provided per area, while moving transect walks will be used to directly assess bumblebee density and morphospecies richness.
3. *Assuring Farm Gate Produce Quality*. Enhancement of nitrogen‐fixation, nutrient application, intercropping and genotype are all expected to impact not only on crude protein content, but on nitrogen: sulphur balance and hence end‐use quality. Seed moisture, size, shape, colour attributes, weight, nitrogen and sulphur content will be measured in all trials.

## WORK PACKAGE 2 – DEVELOPMENT OF PALATABLE, NUTRITIOUS PULSE‐BASED INGREDIENTS AND PRODUCTS

The objective of this work package is to determine how the nutritional value and technological performance of faba bean raw materials can be further enhanced by grain pre‐treatment, milling and product‐making processes. In order to achieve this, three tasks have been designed:

1. *Optimisation of pre‐processing*. The hull reduces milling efficiency of pulses and is rich in tannins, which negatively affect flavour and act as anti‐nutrients (Luo et al., 2010, 2012). The effects of dehulling on grain composition will be determined in combination with a range of thermal and wet processing pre‐treatments (Jiang et al., 2016), focusing on changes in macro‐ and micro‐nutrients (protein, dietary fibre, minerals and selected vitamins), major anti‐nutrients (tannins, lectins and phytates) and precursors of flavour. The biochemical basis for variation in dehulling efficiency will be investigated, aiming to develop rapid screening methods for the selection of this character.
2. *Optimisation of milling and flour characterisation*. Impact of different milling techniques and settings (tempering, break, sizing and reduction systems) on particle size distribution, functional properties (water and oil absorption capacity) and nutrient accessibility (protein and fibre solubility) of flours produced from optimally pre‐treated faba bean will be established (Scanlon et al., 2018). This task will be carried out in strict collaboration with the milling industry and our main focus will be on roller milling, given the better control over particle size (from <100 to 1000 μm) and starch damage achievable with this method. However, we will also test the potential of micronisation (reduction of particle diameter to 50–25 μm) via high‐speed rotary milling for the valorization of the hull and co‐streams produced from roller milling.
3. *Incorporation of ingredients and further processing into bread and other bakery products*. Faba bean flours will be used to produce faba bean‐wheat composite bread (as an exemplar product) at different rates of inclusion. Low percentage inclusions (<3% substitution) will aim to develop faba bean flour functionality as an improver to effectively replace the role currently played by soy in commercial bread‐making, as shown in the preliminary work (Figure 3). Higher inclusion levels (15%–40% substitution) will mainly target improved nutritional quality (protein and micronutrient content/availability). Product physical (texture, cell crumb structure and volume), sensory (quantitative descriptive analysis) and flavour properties, along with acrylamide formation, will be evaluated to optimise the formulation strategy. In collaboration with the industry, test faba bean flours will be used for the development and pilot scale manufacture of cream and snack crackers. Physical, sensory and consumer evaluation of the final crackers will be carried out.

## WORK PACKAGE 3 – DESIGNING HEALTHY, SUSTAINABLE UK GROWN PULSE‐RICH DIETS FOR ALL

The objective of this work package is to determine how UK grown pulses, including faba beans, can be used to produce nutritious, affordable, acceptable and accessible foods such as bread to increase population and environmental health. This will be achieved by analysing and modelling UK dietary data to design and produce optimal dietary patterns prior to the conduct of three human studies. The four specific tasks are briefly described below:

1. *Implications of UK grown pulses on UK diets and the environment*. Existing dietary datasets such as the *National Diet Nutrition Survey* (*NDNS*) and the *UK Biobank* will be used and stratified for appropriate covariates (such as age, sex, ethnicity, geographical location and socio‐economic status), to determine nutritional limitations of current diets and the impact of increased consumption of UK‐grown pulses, including faba beans.
2. *Bioavailability, satiety and functional health outcomes of the pulse‐enriched bread*. Bioavailability of iron and other micronutrients in the ‘Raising the Pulse’ bread will be compared to conventional bread and assessed in a randomised, controlled, suitably powered, cross‐over, postprandial study in adults 18–40 years old which represent a group who commonly have low‐iron status and are the population to be studied in the intervention and some of the consumer studies. Stable isotopes will be used to assess iron absorption from the isoenergetic, iron‐matched test meals containing the pulse enriched and conventional breads. Fasting and postprandial blood samples will be used to measure micronutrients, amino acids, biomarkers of iron status and satiety and CVD risk markers. Self‐reported appetite will also be recorded using visual analogue scales.
3. *Controlled Student Halls Intervention*. A sequential controlled intervention will be performed in two University of Reading full‐board halls of residence. This will be in collaboration with Menus of Change, which is a Global University Research Collaborative that offers a platform for conducting interdisciplinary food systems research and sharing strategies for implementing food‐based health and environmental change in university restaurants and food service operations (https://www.hospitalityuor.co.uk/clever‐cuisine/menusofchange/) and University of Reading catering. In a 9‐week study (3 weeks run‐in, 6 weeks intervention), fresh faba beans, as well as enriched ‘Raising the Pulse’ bread and foods such as flatbreads and hummus co‐designed with consumers and University of Reading catering will be introduced into the menu, both without (first 3 weeks of intervention) and with nutritional, health and environmental impact messages (last 3 weeks of intervention). Students will complete random automated 24‐h recalls and questionnaires to assess diet quality and feelings of satiety. The effectiveness of the impact messages on preferences, acceptability and purchasing of the pulse‐based foods will also be determined.
4. *Campus‐Wide ‘Raising the Pulse’ Campaign*. The Controlled Student Halls Intervention will be extended to include all catering outlets on the main University of Reading campus in collaboration with Menus of Change and University catering. Public, staff and students will engage in an 8‐week communication campaign co‐directed by the British Nutrition Foundation on the nutritional, health and environmental benefits of UK‐grown faba beans and other pulses. Changes in food purchasing behaviour in the University of Reading catering outlets will determine the efficacy of the impact messaging on consumption of ‘Raising the Pulse’ breads and foods, satiety and overall diet quality.

## WORK PACKAGE 4 – CONSUMER ATTITUDES AND BEHAVIOUR TOWARDS PULSES AND CO‐CREATION WITH STAKEHOLDERS

The objective of this work package is to understand consumers' attitudes, preferences and behaviour towards faba bean and pulse products to facilitate market acceptance of the ‘Raising the Pulse’ foods. Different stakeholders' views will be sought during the product development and marketisation phases to identify market challenges and opportunities for wider and timely consumer acceptance of the new faba‐bean enriched products.

1. *Scoping study*. A review of research and stakeholder insights will inform consumer awareness, attitudes, preferences, purchase intentions and consumption for participant profiling and designing the next work package tasks.
2. *New product concept development*. Consumer consultation on current eating habits, the role of pulses, breads and related foods in consumers' diets will help to identify the key factors underpinning consumption of the new products, which will be co‐created with consumers and value chain stakeholders.
3. *Preference ranking of product attributes*. An online survey will identify preferences and inform the final prototype for sensory testing and a discrete choice experiment.
4. *Choice experiment*. Prototype attribute testing will be conducted more widely for determining willingness to pay as well as understanding and predicting consumer acceptance.
5. *Campus surveys*. Our post‐intervention surveys will coordinate with earlier intervention findings of Work Package 3 and will also follow a ‘Raising the Pulse’ communication campaign, in collaborations with Work Package 3, to evaluate impact on consumer awareness and perceptions of key benefits of pulse‐based products and actual food choices.
6. *Test product launch*. The new ‘Raising the Pulse’ foods will be test launched in partner retail outlets (Hodmedod's, Waitrose) to capture wider consumer feedback on product acceptance.

## WORK PACKAGE 5 – SYSTEM PERSPECTIVE

The objective of this work package is to map how the uptake of pulse‐enhanced foods will affect land use, environment, profitability, nutrition, human health and their interactions. There are likely to be benefits for the environment, human nutrition and health, but trade‐offs for product price, with consumer preferences affecting achievable outcomes. For example, a larger percentage of faba bean flour in white sliced bread might improve nutrition, but the uptake of the product and resulting population nutritional outcomes may be greater at a lower faba bean content where the properties appeal to more people. Meanwhile, increasing faba bean percentage will also increase demand for faba beans and decrease demand for breadmaking wheat. Farming practises such as rotations would adjust to this demand, perhaps with faba bean replacing oil seed rape in rotations. A modelling approach is therefore required to consider the complex interactions within the food system due to a change in bread recipe.

1. *Impacts on Land Use and Economics*. An agricultural sector model, LUAM (Tzanopoulos et al., 2012), will be used to consider how different scenarios of market demand for pulse‐enhanced foods and the availability of different pulses for UK production will affect land use, agricultural input use and farm income at farm and national scales. We will also consider implications of different levels of market penetration for pulses, on a scenario basis, through their extension beyond bread into other food products that use flour.
2. *Consequential Life Cycle Assessment*. The environmental impacts of pulse production and processing will be assessed using data from field trials, partner supply‐chain organisations and expert consultation within the supply chain (hauliers, millers and bakers). This collected data and inventory data (https://ecoinvent.org/) will be used with Life Cycle Assessment software (SimaPro) to assess the environmental effects of changes in the levels and type of production and supply.
3. *Understanding causes of yield variability*. A meta‐analytic approach integrating Work Package 1 and stakeholder data with published literature findings will identify the extent and causes of variability in historic faba bean yield and quality across space and time.
4. *Link between nutrition and health*. Data on links between nutrition and health will be collated as pertinent to the replacement of wheat flour, pulse flours (including faba bean) in bread and increase of dietary protein, micronutrients and fibre from pulses, focusing on benefits that can be obtained for key health outcomes such as CVD risk and obesity, and data drawn from extant literature and data from Work Package 3 will be statistically modelled.
5. *Systems model*. A Bayesian Network model will be developed to synthesise findings from across the project, mapping key causal interactions leading to environmental, economic, nutrition and health outcomes. The network structure will be derived from project team expert understanding of the interactions. Model subsections will be parameterised (i.e., the relationships between the different variables in this model and its subsections will be based upon findings from across the project) using project data and meta‐analyses. The Bayesian Network model will be used to identify and consider interventions that could transform the system by improving multiple outcomes.

## IMPACT

As illustrated by the estimates of impact potential for composite faba bean‐wheat bread shown in Figure 3, ‘Raising the Pulse’ will make a significant impact at both the landscape scale, through the conversion of tens of thousands of hectares from wheat to faba bean production, and at the population health scale, through the alleviation of low‐nutrient status and non‐communicable disease risk. Through its industry partnership, ‘Raising the Pulse’ has both capacity and intent to scale up production and marketing of innovative pulse‐enhanced products to capture a significant share of the national bread market. However, to successfully deliver these aims we will need to replicate this feat on a wider geographic scale and across an increased range of food products, and will require us to reach out to, inspire and partner with new stakeholders and partners.

**FIGURE 4:**
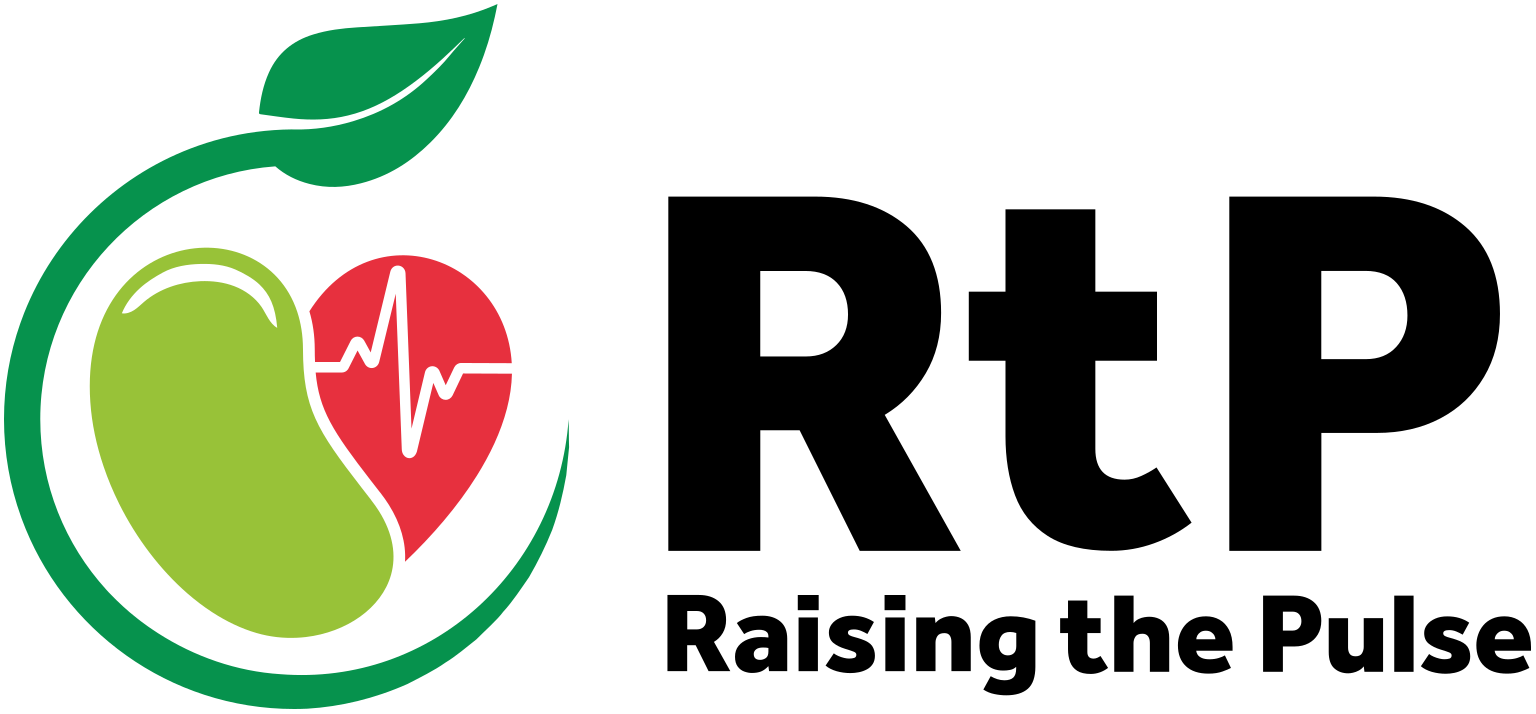
The ‘Raising the Pulse’ project logo. A light green faba‐like bean represents our aim to focus on increasing consumption of pulses. The inclusion of the bean within a red heart superimposed with a pulse trace symbolises how placing pulses like beans and peas ‘at the heart’ of the diet holds the promise of substantial gains in cardiovascular health. The pulse‐bean‐heart icon is encircled by a dark green shoot symbolising how ‘Raising the Pulse’ component of our diet will bring meaningful environmental benefits

To achieve this, annual meetings with relevant stakeholders from the academic, industrial, retail and agricultural communities, in addition to policy makers and non‐government organisations, will be held. These will maximise the impact for knowledge exchange on key outcomes and information with these communities to ensure success in sustainable transformation of the UK food system that will have public health and environmental benefits. Publications and presentations at relevant national and international conferences, including our ‘Raising the Pulse’ symposium to be held as the project concludes, will ensure dissemination of the benefits of a pulse‐enriched foods on sustainable food production and diet quality in collaboration with the British Nutrition Foundation. We have developed a project logo (Figure 4) to give our dissemination materials a visual identity which conveys the core aims and values of the project. Furthermore, collaboration and knowledge‐exchange with existing BBSRC Transforming Food Systems projects will maximise and enrich the outcomes generated from these individual projects.

It is of note that the majority of faba beans grown in the United Kingdom are used for animal feed. Much of the insight into the economic and environmental optimisation of faba bean production systems and at least some factors found to impact quality and initial processing steps can be exploited to the benefit of the animal feed industry. However, as the transformative objective behind 'Raising the Pulse' research is to facilitate wider acceptance and more direct human consumption of plant protein, rather than indirect consumption of plant protein converted inefficiently into animal protein, we will not explore these avenues as part of this project.

## ‘Raising the Pulse’ Team

Members of the 'Raising the Pulse' team from University of Reading include: ***WP1*** – Professor Donal M O'Sullivan (lead), Professor John Hammond, Professor Elizabeth Shaw; ***WP2*** – Dr Paola Tosi (lead), Dr Afro Chatzifragkou, Dr Stella Lignou, Dr Jane K Parker, Dr Julia Rodriguez‐Garcia; ***WP3*** – Professor Julie A Lovegrove (PI and lead), Dr Miriam E Clegg, Dr Kim G Jackson, Professor Paul Sharp (Kings College London), Matt Tebbit (University of Reading, Menus of Change); ***WP4*** – Dr Elena Millan (lead), Dr Anna L Macready; ***WP5*** – Dr Jacob Bishop (lead), Philip J Jones, Dr Laurence G Smith, Dr Lindsay C Todman. Project Co‐ordinator – Yvonne McMeel. Project partners include Professor Richard Tiffin (Agrimetrics Ltd), Phil Metcalfe (BioPower Technologies), Dr Sarah Stanner (British Nutrition Foundation), Dr Helen Riordan (DEFRA Westminster), Dr Mervin Poole (Heygates Ltd), Josiah Meldrum (Hodmedod Ltd), Bruce Knight (Legume Technology Ltd), Roger Vickers [Processors and Growers Research Organisation (PGRO)], Dr Emma Williams, Nathalie Winn, Rebecca Hesketh (Waitrose), Dr David A Garrec and Dr Jennifer Moss (Pladis (UK).

## FUNDING INFORMATION

The *‘Raising the Pulse’ (RtP): Systems analysis of the environmental, nutritional and health benefits of pulse‐enhanced foods* project (BB/W017946/1) received funding from the UK Research & Innovation (UKRI) Biotechnology and Biological Sciences Research Council (BBSRC) in the Transforming UK Food System SPF Programme – Call 2.

## CONFLICT OF INTEREST

There are no conflicts of interests to declare.

## Data Availability

The data that support the findings of this study are available from the corresponding author upon reasonable request.
